# SYL3C aptamer-anchored microemulsion co-loading β-elemene and PTX enhances the treatment of colorectal cancer

**DOI:** 10.1080/10717544.2019.1660733

**Published:** 2019-09-14

**Authors:** Xiaorong Zhou, Chuanpei Cao, Nan Li, Shaofei Yuan

**Affiliations:** a Department of Medicine, Jiangsu Cancer Hospital, Nanjing, People’s Republic of China;; b Gastrointestinal Surgery, Affiliated Hospital of Jiujiang University, Jiujiang, People’s Republic of China;; c Department of Medical Oncology, The Third Affiliated Hospital of Wenzhou Medical University, Wenzhou, People’s Republic of China

**Keywords:** PTX, β-elemene, colorectal cancer, AS1411 aptamer, EpCAM

## Abstract

The aim of this study is to construct a SYL3C aptamer-anchored microemulsion based on β-elemene and PTX (SYL3C/EP-MEs) for enhancement on colorectal cancer therapy. Such microemulsion is consist of encapsulated drugs (β-elemene and PTX), tumor targeting ligand (3’-end thiolated SYL3C aptamer), thiol conjugated site (maleimide-modified PEGylated 1,2-dioleoyl-sn-glycero-3-phosphoethanolamine, mal-DOPE-PEG), pH-sensitive component (DOPE) and other necessary excipients. SYL3C/EP-MEs showed a spherical particle with an average particle size around 30 nm and a high encapsulation efficiency (>80%) for both drugs. β-elemene and PTX could be released controllably from SYL3C/EP-MEs as pH values changed. SYL3C/EP-MEs displayed a selective affinity to HT-29 cells, leading to an obvious increase in cellular uptake, cell apoptosis and cytotoxicity. In the HT-29 tumor xenograft-bearing nude mice model studies, SYL3C/EP-MEs showed an overwhelming tumor growth inhibition, the longest survival time and the lowest systemic toxicity among all the treatments. The potential mechanism of enhanced anti-cancer ability was probably associated with the induction of M1 macrophage polarization, the downregulation of mutant p53 protein and the reduction of bcl-2 protein expression. Collectively, the microemulsion codelivery of β-elemene and PTX using functionalization with SYL3C aptamer provides a novel approach for combinational colorectal cancer-targeted treatment.

## Introduction

The China National Cancer Center (CNCC) recently released a cancer-related report that states colorectal cancer is still one of the most frequently occurring malignant tumors in China. Its incidence rate is 9.88%, ranking it the third place after lung and gastric cancer, while its mortality rate is 8.00%, ranking it the fifth place after lung, liver, stomach, and esophageal cancer (Sung et al., 2005; Gu et al., 2018). Moreover, China’s colorectal cancer-related mortality rate is higher than that of western countries, owing to limited early diagnostic and treatment techniques, although its incidence rate is lower than that of Europe and the US (Brenner et al., 2014; Meester et al., 2015). Combination chemotherapy, surgical resection, and radiotherapy are the most popular colorectal cancer treatments. As reported, post-surgical FOLFOX combination chemotherapy (oxaliplatin, leucovorin, and 5-fluorouracil) and “sandwich” (FOLFOX-radiotherapy-FOLFOX) combination therapy significantly prolong patients’ overall survival (Bokemeyer et al., 2011; Cassidy et al., 2011; Xiao et al., 2015). However, conventional chemotherapeutics are poorly distributed, causing severe gastrointestinal, liver, and kidney toxicity, immunosuppression, and other side effects, leading to treatment termination, which affects the overall treatment effect (Huang et al., 1998; Fujii et al., 2003). Therefore, a rational drug combination and an efficient tumor-targeting treatment method, with fewer side effects but improved anti-tumor activities, are urgently needed.

β-elemene, a non-cytotoxic broad-spectrum anti-tumor component extracted from traditional Chinese medicine, has been clinically used as adjuvant treatment for lung, gastric and colorectal cancers (Wang et al., 2005; Jing et al., 2011), although its anti-tumor mechanism remains unknown. It reportedly down-regulates the expression of the bcl-2 and survivin genes, inhibits the activity of telomerase and the mutant P53 activating fragment, and up-regulates the expression of the bax and PDCD5 genes, to induce or promote tumor cell apoptosis (Jiang et al., 2016). Also, it can sensitize various chemotherapeutic agents and improve their anticancer efficacy. For example, β-elemene was shown to enhance the sensitivity of lung cancer cells to cisplatin by down-regulating the expression of bcl-2 and inhibitor of apoptosis proteins (IAPs) (Li et al., 2009, 2010). We previously showed that the combination of β-elemene and PTX significantly improves their individual anti-proliferative effect on colon cancer HT-29 and LoVo cells, although the underlying mechanism remains unclear. The results of this study also showed that β-elemene did not reduce the cytotoxicity of PTX in normal cells, suggesting the need for targeted drug delivery via a drug delivery system, which will improve the efficacy and reduce the side effects of the combination therapy. Zhang et al. recently developed a β-elemene/celastrol bicomponent microemulsion system which to some extent efficiently delivers combination cancer drugs to target sites, enabling an increased synergistic anticancer effect via nanoparticle-associated passive drug accumulation in tumors (Zhang et al., 2019). This inspired the design of a β-elemene-based codelivery system in this study.

The transmembrane glycoprotein epithelial cell adhesion molecule (EpCAM), highly expressed in epithelial cancers, is a popular biomarker for the diagnosis, treatment, and imaging of epithelial-derived tumors such as colorectal cancer (Munz et al., 2009). Currently, monoclonal antibody probes are the most popular clinical diagnostic tools and the most validated immunostaining method for detecting EpCAM; however, their application is limited by their high production cost (Stott et al., 2010). Aptamers are repeatedly screened from a library of random oligonucleotide sequences synthesized in vitro, to bind to target molecules with extremely high affinity and specificity through a systematic evolution of ligands by exponential enrichment (SELEX) technique, and can be based on RNA, single-stranded DNA, or double-stranded DNA (Sampson, 2003). Aptamer binding to target molecules is similar to antigen-antibody binding, and has the advantage of high affinity and specificity to the ligand. Compared to antibodies, it is safe, less expensive, and simple to prepare (Kim & Man, 2014). However, a previously developed RNA-based EpCAM aptamer is reportedly easily degraded by nucleotidase in vivo, inhibiting its practical application (Shigdar et al., 2011). Notably, the more recently designed DNA-based EpCAM aptamer SYL3C, has better stability and is more cost-effective than previously designed RNA-based aptamers (Song et al., 2013). SYL3C was capable of specifically targeting, labeling, and binding to colorectal tumor cells, acting not only as an alternative to antibodies, but also as a promising potential tumor-targeting ligand. However, to date, there are few reports about its applications in tumor-targeting nano-sized drug delivery systems.

Based on previous progress, we integrated β-elemene and PTX into a microemulsion drug delivery system, with the tumor-targeting ligand SYL3C anchoring on the surface of the particles. To overcome the uncontrollable drug release associated with conventional microemulsion drug delivery systems, we employed commonly-used pH-sensitive lipid vector 1,2-dioleoyl-sn-glycero-3-phosphoethanolamine (DOPE) (Briscoe et al., 1995) as a part of the mixed surfactant. To modify microemulsion with SYL3C aptamer, maleimide-modified PEGylated 1,2-dioleoyl-sn-glycero-3-phosphoethanolamine (MAL-PEG-DOPE) was incorporated into microemulsion to stably conjugate with sufficient 3’-end of thiolated aptamers, through a specific Michael addition reaction between α,β-unsaturated ketone double bonds and thiol groups (Jiang et al., 2015). This study focused on the optimization and characterization of the preparation, pH-sensitive drug release, in vitro tumor targeting and synergistic anti-tumor effects, in vivo anti-tumor efficacy, and preliminary molecular mechanisms, to rationalize the effectiveness of our dual component-based, tumor-targeting microemulsion drug delivery system design.

## Materials and methods

### Materials

β-elemene was purchased from Nanjing Axianu BioTech Co., Ltd. (Jiangsu, China); Paclitaxel (PTX) was bought from China Pharmaceutical Group (Shanghai, China); Thiolated oligonucleoide DNA aptamer (SYL3C) with the following sequence as 5′-CAC TAC AGA GGT TGC GTC TGT CCC ACG TTG TCA TGG GGG GTT GGC CTG-(PEG)_3_-SH-3’ was purchased from Sangon Biotech Co., Ltd. (Shanghai, China); Kolliphor HS15 (HS15) was offered by BASF Co., Ltd. (Germany); Labrafil M 1944CS (1944CS), fluorescin isothiocyanate (FITC) and PEG 400 were provided by Aladdin Bio-Chemo Tech Co., Ltd. (Shanghai, China); DOPE and Mal-PEG-DOPE were purchased from Lipoid GmbH Co., Ltd. (Germany); Dulbecco's Modified Eagle Media (DMEM), Roswell Park Memorial Institute (RPMI) 1640 medium, F-12K medium, fetal bovine serum (FBS), 3-(4,5-dimethyl-2-thiazolyl)-2,5-diphenyl-2-H-tetrazolium bromide (MTT) were purchased from Thermo-Fisher Co., Ltd. (Beijing, China). Terminal deoxynucleotidyl transferase dUTP nick end labeling (TUNEL) kit, Antihuman-EpCAM mouse monoclonal anti body MOC-31, anti-P53 antibody, anti-CD68 antibody, anti-Bcl-2 antibody and FITC-labeled secondary antibody were bought from Abcam BioTech Co., Ltd. (Cambridge, MA). IFN-γ and IL-10 ELISA kits were purchased from KeyGene BioTech Co., Ltd. (Jiangsu, China).

### Optimization on microemulsion preparation

SYL3C/EP-MEs was prepared by one-step emulsion method as described previously but with some modifications (Qu et al., 2014, 2015; 2017; Su et al., 2017; Zhang et al., 2019). First, 4 ∼ 40 mg of PTX and 80 mg of β-elemene were simultaneously dissolved in 220 mg of 1944 CS with a vigorous stirring for 2 h. Next, 170 mg of DOPE, 120 mg of HS15 and 10 mg of Mal-DSPE-PEG were added to the above-mentioned homogeneous mixture in batches. After further 2 h of strong stirring, 100 mg of PEG400 was added and stirred until complete homogeneity. And then, 5.0 mL of deionized water was dropwise dropped into the resultant mixture to gain a clear and transparent microemulsion (EP-MEs). As for the preparation of SYL3C/EP-MEs, 250 nM of SYL3C dissolved in 0.3 mL of water was incubated with prepared EP-MEs solution for 24 h at room temperature. The unconjugated aptamers were removed after purification using G-50 sephadex column.

### Characterization of microemulsions

The main physicochemical parameters of SYL3C/EP-MEs were measured by a dynamic light scattering (DLS) (Zetasizer Nano ZS90, Malvern, UK). Various samples diluted as 1 mg/mL (total mass concentration) were injected into the sample cell to obtain the average size, polydispersity (PDI) distribution and zeta potential according to the protocol of operation (Su et al., 2017). As reported previously, the morphology was characterized by transmission electron microscopy (TEM, JEOL-100CXII, Japan) (Qu et al., 2014; 2017). Briefly, 10 μL of SYL3C/EP-MEs at a concentration of 20 mg/mL (total mass concentration) was dropped onto a TEM-exclusive copper grid for 5 sec. At the end of the deposition, the film was stained with 1% (wt%) phosphotungstic acid for 10 sec. And then, the grid with sample was observed immediately by TEM after air-dry.

The entrapment efficiency (EE) of PTX in microemulsions was calculated by the following equations: EE (%) = (C_test_×V_test_/W_feeding_) ×100%, C_test_, V_test_, and W_feeding_ represents the PTX concentration of the sample, the volume of the microemulsion, and the total feeding PTX, respectively. The HPLC chromatographic condition of PTX is as follows, column:  Inersil®ODS-3 C18 (4.6 mm × 150 mm, 5 µm); flow rate: 1.0 mL/min; detection wave: 227 nm; mobile phase: methanol/water = 65/35; column temperature: 35 °C; and injected sample volume: 20 µL (Qu et al., 2013).

### Pseudoternary phase diagrams

As reported previously, β-elemene and 1944CS were used as the mixed oil, HS15, DOPE and Mal-PEG-DOPE were employed as the mixed surfactant, and PEG 400 was selected as the cosurfactant (Zhang et al., 2019). The weight ratio of HS to DOPE (and Mal-PEG-DOPE) was predetermined as 2:1, 1:1 and 2:3, and the mixed surfactant was named as ^1^S_mix_, ^2^S_mix_, ^3^S_mix_, respectively. In the preparation of microemulsion, the K_m_ calculated by a mass ratio of surfactant to cosurfactant was set as 2:1. According to the similar above-mentioned preparation procedures, formulation appearing a clear and transparent solution was regarded as a microemulsion, and that displaying a gel-like semisolid state was considered as a gel. The microemulsion zone, as well as gel zone, was demarcated through connecting the corresponding coordinates in the pseudoternary phase diagrams.

### In vitro *drug release*

The PTX release profile of microemulsions was measured by a modified dialysis method (Ruan et al., 2012). 5 mL of EP-MEs and SYL3C/EP-MEs packed in the dialysis bag (molecular weight cutoff, MWCO, 5 kDa) were placed in a dissolution apparatus (VFS-IR, Henan, China), followed by immersion in 250 mL of phosphate buffer saline (PBS) with various pH values (5.0, 6.5 and 7.4) at 37 °C under a stirring at 90 rpm, respectively. At each predetermined time intervals (0 ∼ 48 h), 0.5 mL of dissolution medium was withdrawn to detect the content of PTX by HPLC. *In vitro* PTX release was calculated by the following formulas, release (%) = C_PTX_ × 0.5 × 250/M_PTX_ × 100%, where C_PTX_ and M_PTX_ represents the HPLC-detected PTX concentration of each sample and the initial amount of PTX in microemulsions.

### Serum stability of microemulsion

One milliliter of SYL3C/EP-MEs containing 100 µg/mL PTX was incubated with equivalent FBS for 12 h at 37 °C. During the period of the observation, the particle and zeta potential of microemulsions was recorded at the predetermined intervals. Likewise, the PTX leakage from SYL3C/EP-MEs was detected by HPLC as the following formula, leaking PTX (%) = 100% − (PTX _in microemulsion_/PTX _feeding_) × 100%.

### Cells culture

Two types of human colorectal tumor (HT-29 and Lovo) cells purchased from American Type Culture Collection (ATCC) were cultured in F-12K and DMEM medium, respectively, supplemented with 10% (v%) FBS, 100 U/mL penicillin and 100 μg/mL streptomycin. The normal colonic epithelial (NCM460) cells were cultured in RPMI 1640 medium containing 10% of FBS, 100 U/mL penicillin and 100 μg/mL streptomycin. Cells were incubated in a cell incubator (Thermo 3110, USA) with an atmosphere of 5% CO_2_ at 37 °C.

### Cellular immunostaining by anti-EpCAM antibody

A hundred thousand of HT-29 cells and NCM460 cells were seeded in 12-well plates embedded a polylysine-coated glass sheet for 24 h, respectively. According to the protocol of EpCAM antibody staining, the cell-loaded slide was incubated with 0.1% Triton X-100 and blocked with 1% BSA for 30 min, successively. Next, the cells were conjugated with 200-fold diluted primary monoclonal anti body MOC-31 (Abcam, UK) for 1 h at room temperature. After washing thrice with PBS, the cells were stained with 200-fold diluted FITC-conjugated secondary antibody for 1 h, followed by washing with ice-cold PBS thrice. After further staining with DAPI for 30 min, the cells were finally fixed with 4% paraformaldehyde for 15 min (Ying et al., 2015). The immunostaining images were acquired immediately by confocal laser scanning microscopy (FV101i, OLYMPUS, Japan) using binary channels. All the operations are performed at room temperature.

### Intracellular fluorescence of FITC-labeled microemulsions

FITC-labeled EP-MEs (FITC/EP-MEs) and FITC-labeled SYL3C/EP-MEs (FITC/SYL3C/EP-MEs) were prepared by the above-mentioned microemulsion preparation method after incorporation with 0.05% (wt%) of FITC, and the mixture of equivalent β-elemene, PTX and FITC (β-elemene + PTX + FITC) was used as the control group. 1 × 10^6^ of HT-29 cells were cultured in 6-well plates overnight. After adherence, the cells were incubated with 5 μM of β-elemene + PTX + FITC, FITC/EP-MEs, FITC/SYL3C/EP-MEs and SYL3C (250 nM, 0.5 h)-pretreated FITC/SYL3C/EP-MEs for 4 h, respectively. At the end of the treatment, the cells were rinsed by PBS and acquired the fluorescence images by a fluorescence inverted microscope (IX73, Olympus, Japan) immediately (Ming et al., 2016).

### Quantification of intracellular PTX

A hundred thousand of HT-29 cells were seeded into 12-well plates and cultured in a cell incubator until complete adherence. Next, the cells were treated with the following formulations, (1) β-elemene + PTX (8/1, w/w), (2) EP-MEs, (3) SYL3C/EP-MEs and (4) SYL3C (250 nM, 0.5 h)-pretreated FITC/SYL3C/EP-MEs, for 4 h at a PTX concentration of 20 µg/mL. After the treatments, the cells were washed with PBS and lysed with 150 µL of 0.1% (wt/%) of sodium dodecyl sulfate (SDS) for one minutes. Intracellular PTX was extracted from 100 µL of cell lysate by methanol and detected by HPLC. The cell protein was quantified through a BCA protein assay kit. The intracellular PTX (µg/mg) was calculated as the ratio of intracellular PTX content to the amount of cell protein (Qu et al., 2013).

### Cell viability assay

Five thousand of HT-29 cells, as well as Lovo cells, were seed into in 96-well culture plates. After reaching 60% of confluence, the cells were treated with (1) β-elemene + PTX (8/1, w/w), (2) EP-MEs, (3) SYL3C/EP-MEs and (4) SYL3C (250 nM, 0.5 h)-pretreated FITC/SYL3C/EP-MEs, respectively, at PTX concentrations ranging from 0.01 ∼ 10.0 µg/mL. After 24 h of treatment, the cells were stained with 5 mg/mL of MTT for 4 h, followed by dissolving with 160 μL of dimethyl sulfoxide (DMSO). A microplate reader (SpectraMax iD5, MD, USA) was used to record the absorbance at 490 nm. The cell viability (%) was calculated as the ratio of the absorbance of test group to the absorbance of control group (Yi et al., 2012). The half-maximal inhibitory concentration (IC50) was reckoned by SPSS16.0 software. The combined index (CI) was calculated as the following formula, CI = ^a^IC50_a+b_/IC50_a_ + ^b^IC50_a+b_/IC50_b_, where IC50_a_ and IC50_b_ represent the IC50 of a-used alone and b-used alone against tumor cells, respectively, ^a^IC50_a+b_ and ^b^IC50_a+b_ represent the IC50 of the combined treatment against tumor cells calculated in a and b, respectively (Kaufmann et al., 1996).

### Tumor-bearing animal model

BALB/c nude mice with the weight around 22 g were purchased from Model Animal Research Center of Nanjing University (Jiangsu, China). The mice were bred in accordance with the protocol approved by the animal ethics committee of our hospital. After 7 day of feeding, 2 × 10^7^ of HT-29 cells was subcutaneously injected into the right back to prepare the HT-29 tumor xenograft-bearing nude mice model (Pearson et al., 1989).

### *Antitumor efficacy and pathological section* in vivo

When the tumor volume reached 60 ∼ 80 mm^3^, sixty mice were randomly divided into five groups, and then intravenously injected with (1) β-elemene + PTX (8/1, w/w), (2) EP-MEs and (3) SYL3C/EP-MEs at a PTX dose of 10 mg/kg, respectively. For aptamer competition test, the mice were injected with 20 µL of SYL3C (250 nM) in the tumor in situ, followed by treating with SYL3C/EP-MEs after 6 h. Saline was used as the negative control group. During the treatments, the tumor size, body weight and survival period were recorded every day. At the end of the treatment, the tumor, liver and spleen of mice were collected and weighted, respectively. The organ index was calculated as a ratio of the organ weight to the body weight. Besides, the ex vivo tumor tissues were prepared into for the following hematoxylin and eosin (HE), immunohistochemical (IHC, including Ki-67, p53, CD86 and bcl-2) and terminal deoxynucleotidyl transferase dUTP nick end labeling (TUNEL) staining, using a fluorescence microscope (IX73, Olympus, Japan) (Ratnasinghe et al., 1999). The quantification of positive cell ratio of various IHC images was calculated by Image J software.

### Cytokines and biochemical indictors

After 12 h of the last administration, 200 µL of blood was collected from four mice selected randomly from each of group, followed by preparing serum sample. To evaluate the liver and kidney function, an automatic biochemical instrument (AU5800, Beckman Coulter, USA) was used to detect several main biochemical indictors, including aspartate transaminase (AST), alanine transaminase (ALT), blood urea nitrogen (BUN) and creatinine (CREA). Likewise, after 24 h of the last administration, 100 µL of serum was prepared to detect interferon-gamma (IFN-γ) and interleukin-10 (IL-10) by corresponding enzyme-linked immunosorbent assay (ELISA) kits, according to the experimental protocol (Qu et al., 2018).

### Data analysis

In this study, all the showed data represent mean ± standard deviation. Two-tailed Student’s t test was employed to perform statistical tests. *p* < .05 and *p* < .01 represent a significant and an extremely significant difference, respectively.

## Results and discussion

### Characterization of microemulsions

**Figure 1.**
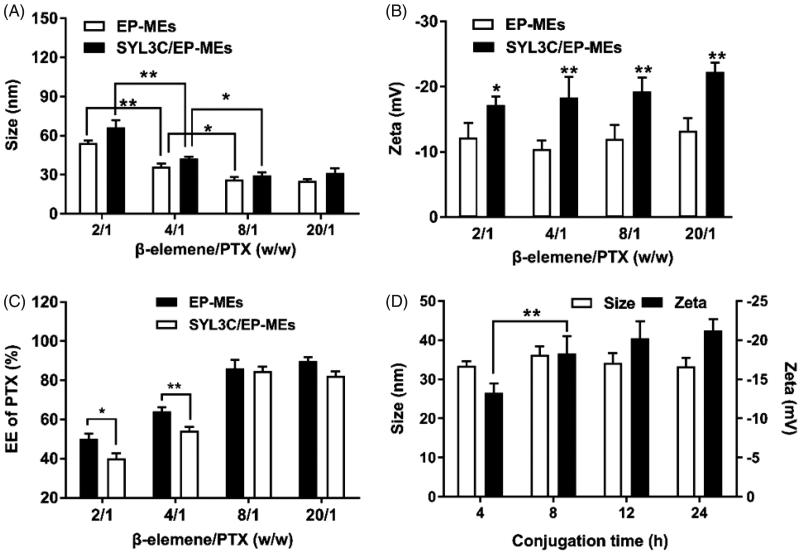
Optimization of preparation technology. Influence of different weight ratios of β-elemene to PTX on (A) particle size, (B) zeta potential and (C) PTX encapsulation efficiency of two types of microemulsion. *n* = 4, *p < .05, **p < .01. (D) Influence of different conjugation time on the particle size and the zeta potential of SYL3C/EP-MEs. *n* = 4, **p < .01.

In this study, β-elemene and 1944CS were used as the mixed oil phase; HS15, DOPE and Mal-DOPE-PEG were employed as the mixed surfactant; and PEG-400 was incorporated into microemulsions as a cosurfactant. As documented in the previous papers (Qu et al., 2017; Zhang et al., 2019), the optimal mass ratio of oil phase to surfactant was 3/4, and the feeding ratio of surfactant to cosurfactant (K_m_) was 3/1 in such type of microemulsion. Optimizing the mass ratio of the 2 payloads in a dual component-based delivery system is a well-known challenge. Based on our previous studies, the mass ratio of β-elemene to PTX is one of the most critical factors affecting the characteristics of SYL3C/EP-MEs and EP-MEs. Here, the mass ratio of β-elemene to surfactant was determined as 4/15, and that of β-elemene to PTX was regulated by adjusting the feeding of PTX. As shown in Figure 1(A), the particle size of SYL3C/EP-MEs was 66.3 ± 5.4 nm when the mass ratio of β-elemene to PTX was 2/1. With the mass ratio increased, the particle size of both microemulsions decreased significantly, until up to 8/1, suggesting that the particle size of the microemulsions were smallest when the mass ratio was higher than 8/1. A similar trend was also observed with the particle size of EP-MEs. Figure 1(B) shows that the β-elemene to PTX mass ratios did not significantly influence zeta potential; however, the zeta potential of SYL3C/EP-MEs is obviously lower than that of EP-MEs at their corresponding mass ratios. This could be a result of the negative charge of the DNA-based SYL3C aptamer modified outside the microemulsion (Masuda et al., 2016). As shown in Figure 1(C), the EE of PTX was significantly different with various mass ratios. When the β-elemene to PTX mass ratio was 2/1, the EE of PTX in both microemulsions was lower than 60%, probably owing to PTX overload. When the mass ratio increased to 8/1, the EE of PTX in both microemulsions was higher than 80%. Notably, the EE of PTX in both microemulsions showed statistically significant difference at low mass ratios, probably because of the rapid leakage of overloaded PTX from SYL3C/EP-MEs during aptamer modification. Besides, because β-elemene was a part of the oil phase in the microemulsion system, its EE was not detected in this study. Another important factor affecting the surface properties of the microemulsions was the reaction time of thiol-SYL3C aptamers and maleylated microemulsions. As shown in Figure 1(D), their zeta potential did not continue to decrease after the reaction time exceeded 8 h, suggesting that the conjugation of SYL3C and microemulsion reached its plateau stage. Therefore, the reaction time of the thiolated SYL3C and mal-modified microemulsion was set as 8 h.

### Pseudoternary phase diagrams

**Figure 2.**
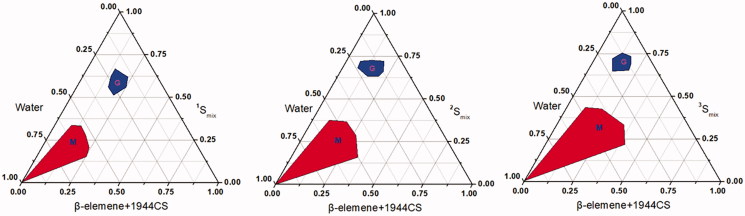
Pseudoternary phase diagrams of SYL3C/EP-MEs at different mass ratio of DOPE&Mal-DOPE-PEG to HS15. M zone represents the area of microemulsion, and G zone represents the area of gel, respectively. ^1^S_mix_, ^2^S_mix_, ^3^S_mix_ represent the mass ration of DOPE&Mal-DOPE-PEG to HS15 as 13/7, 11/9, 9/11, respectively.

In this study, HS15, DOPE, and Mal-DOPE-PEG were used as the mixed surfactants. Based on the results of our previous studies, mutual mass ratio is an important factor affecting the formation of microemulsions. Here, the K_m_ and mass ratio of mixed oil to mixed surfactant were determined to be 1/3 and 3/4, respectively. We then optimized the mass ratio of DOPE&Mal-DOPE-PEG to HS15 via the pseudo-ternary phase diagram. As shown in Figure 2, the microemulsion prepared with the HS15 and DOPE&Mal-DOPE-PEG mass ratio of 9/11 exhibited the largest formation area among the 3 groups, suggesting that the mixed surfactant with such mass ratio is easiest to use for microemulsion formation. In summary, the optimal mass ratio of PTX/β-elemene/DOPE/Mal-DOPE-PEG/HS15/1944CS/PEG400 was determined as 10/80/170/10/120/220/100 (see Table 1).

**Table 1.** Optimized constituents of EP-MEs and SYL3C/EP-MEs.

Formulation	SYL3C (nM)	PTX (mg)	*β*-elemene (mg)	DOPE (mg)	Mal-DOPE-PEG (mg)	HS15 (mg)	1944CS (mg)	PEG400 (mg)
EP-MEs	NA	10	80	180	0	120	220	100
SYL3C/EP-MEs	250	10	80	170	10	120	220	100

### pH sensitivity of microemulsions

**Figure 3.**
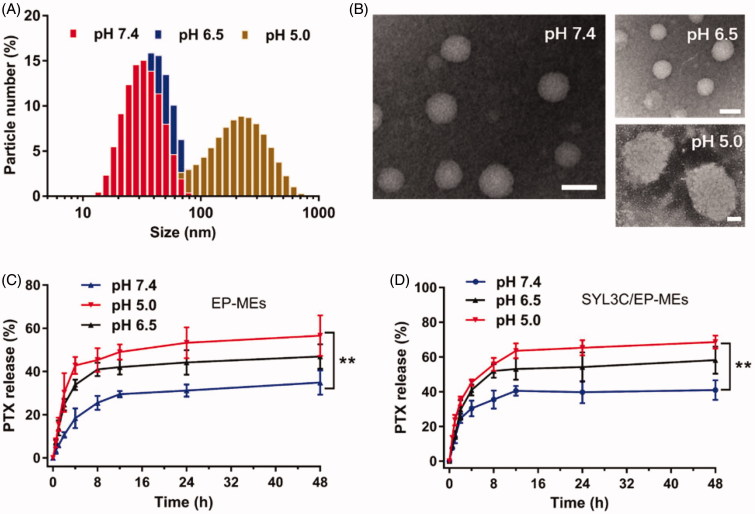
Evaluation on pH sensitivity of microemulsions. (A) Distribution of particle size and (B) morphology of SYL3C/EP-MEs at different pH values. The bar is 50 nm. Accumulative release of PTX from (C) EP-MEs and (D) SYL3C/EP-MEs at different pH values. *n* = 4, **p < .01.

In this study, DOPE, a commonly-used pH-sensitive lipid, was employed as a part of a mixed surfactant to achieve pH-responsive release properties for the microemulsions. As shown in Figure 3(A), the particle sizes of SYL3C/EP-MEs at pH 7.4 and 6.5 were both approximately 30 nm. However, as the pH decreased, the particle size sharply increased (up to approximately 300 nm). Figure 3(B) displays the morphology of SYL3C/EP-MEs at various pH values. In a mildly acidic environment, its shape was spherical and its particle size was uniformly distributed. Microemulsion particle size rapidly increased under acidic conditions, which was in accordance with the DLS results. The potential mechanism is probably associated with the pH being lower than the pKa of DOPE, leading to protonation and lipid rearrangement, and thus, significant swelling of the hydrophilic layer of the microemulsions (Cheng, 1990). To verify this hypothesis, we examined microemulsion release behavior at different pH. As shown in Figure 3(C,D), drug release from both microemulsions showed obvious pH sensitivity. The 48 h-cumulative release of both microemulsions reached approximately 60% at pH 5.0. In comparison, the both microemulsions released only 30% of PTX at pH 7.4, suggesting that DOPE possessed similar characteristic in our microemulsion systems, apart from the ability to achieve a pH-sensitive drug release in liposomal systems (Shin et al., 2012). Besides, the modification of SYL3C did not significantly affect its drug release profile.

### Stability of microemulsions

**Figure 4.**
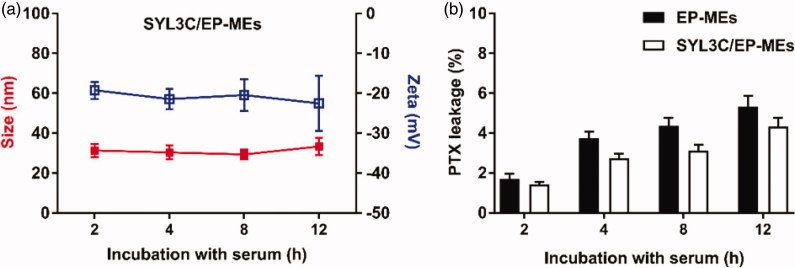
Stability of microemulsions. (A) Particle size and zeta potential of SYL3C/EP-MEs after incubation with serum for different time intervals. (B) Leakage of PTX from SYL3C/EP-MEs at different time post incubation with serum.

In vivo stability is well known to be one of the prerequisites for microemulsions’ targeted delivery and low systemic toxicity. We therefore examined the pharmaceutical behavior changes of SYL3C/EP-MEs after 12 h of incubation with serum. As shown in Figure 4(A), there was no obvious change in the particle size and zeta potential of SYL3C/EP-MEs. Similarly, the leakage rate of PTX was controlled below 10% at 12 h post incubation with serum (Figure 4(B)). These results suggest the relatively stability and low drug leakage of SYL3C/EP-MEs during blood circulation, elucidating its benefits in tumor targeting and side effect reduction.

### Cellular studies

**Figure 5.**
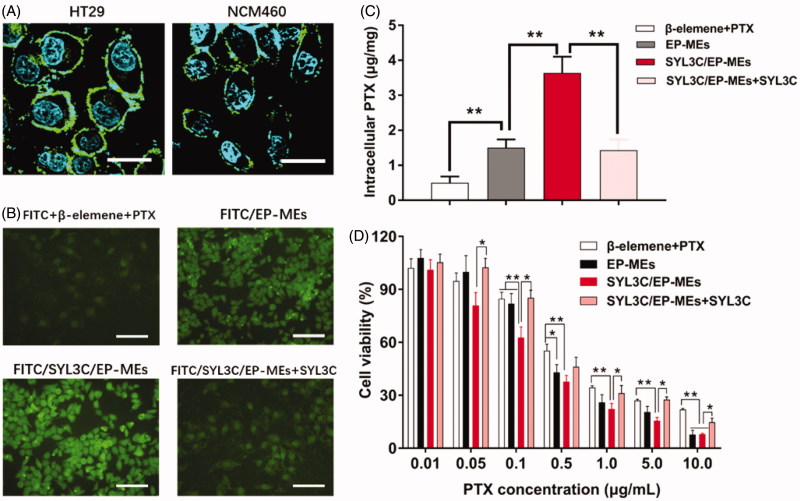
Cellular studies. (A) Immunofluorescence staining of HT-29 cells and NCM460 cells by anti-EpCAM antibody. The bar is 50 μm. (B) Intracellular fluorescence of HT-29 cells after treatments with different FITC-labeled formulations for 4 h. The bar is 200 μm. (C) Intracellular PTX of HT-29 cells after treatments with different PTX formulations for 4 h. *n* = 4, **p < .01. (D) Cytotoxicity of different formulations against HT-29 cells for 24 h. *n* = 6, *p < .05, **p < .01.

The anti-EpCAM antibody was used for immunofluorescent staining of colon cancer HT-29 cells and normal colonic epithelial NCM460 cells, to validate the significant difference in their expression of EpCAM. As shown in Figure 5(A), the green fluorescence signal was obviously observed on the surface of HT-29 cells, but weakly found on the surface of NCM460 cells, suggesting that SYL3C selectively enabled SYL3C/EP-MEs internalization by HT-29 cells during absorption in the colon. Next, we investigated the influence of SYL3C modification on the cellular uptake of HT-29 cells. The results showed that the intracellular fluorescence of FITC/EP-MEs was significantly improved compared with the physically mixed group, showing the inherent cellular uptake advantage of the microemulsion system. After the modification with SYL3C, the intracellular fluorescence intensity of FITC/SYL3C/EP-MEs further improved (Figure 5(B)). However, pretreatment with 250 nM of SYL3C for 0.5 h significantly decreased the cellular uptake of FITC/SYL3C/EP-MEs, indicating that cellular uptake enhancement was associated with the microemulsion system and SYL3C modification. With regards to quantitative internalization, intracellular PTX content after incubation with SYL3C/EP-MEs was 3.64 ± 0.45 μg/mg, 2.21 and 1.14 folds higher than that of the β-elemene + PTX and EP-MEs groups, respectively (Figure 5(C)). However, the increased cellular uptake was significantly attenuated after competitive inhibition by SYL3C aptamer pretreatment. Further, we evaluated the cytotoxicity of various PTX formulations in HT-29 cells. As shown in Figure 5(D), when the PTX concentration was above 0.5 μg/mL, the cytotoxicity of the EP-MEs group was significantly enhanced compared with that of the physically mixed group. As expected, SYL3C pretreatment ameliorated the SYL3C/EP-MEs-induced elevated cell growth inhibition. In addition, we evaluated the synergistic effect of SYL3C/EP-MEs in two types of colon cancer (HT-29 and LoVo) cells. The CI of the β-elemene + PTX group was 0.99 after incubation with HT-29 cells for 24 h (Table 2). Once both drugs were co-loaded into the microemulsion, the CI of the EP-MEs group decreased to 0.73. When SYL3C was incorporated, the CI of the SYL3C/EP-MEs group further reduced to 0.63, suggesting the sufficient transported of both drugs into the tumor cells was beneficial to their synergistic anticancer effect. β-elemene + PTX showed no synergistic anti-tumor effect after 24 h of incubation with LoVo cells (Table 2). However, a synergistic effect was found in the EP-MEs-treated group. The CI of SYL3C/EP-MEs further decreased to 0.87, validating the positive effects of codelivery and uptake enhancement on the synergistic anticancer effect in vitro (Zhang et al., 2019).

**Table 2.** IC_50_ and CI of formulations against two types of colorectal cancer cells (*n* = 6, ± SD).

Formulation	HT-29 (μg/mL)	CI	Lovo (μg/mL)	CI
β-elemene	61.23 ± 2.71	NA	67.84 ± 3.20	NA
PTX	0.96 ± 0.08	NA	1.06 ± 0.12	NA
β-elemene + PTX	0.85 ± 0.04**	0.99	0.95 ± 0.04**	1.01
EP-MEs	0.63 ± 0.03**	0.73	0.89 ± 0.03**	0.95
SYL3C/EP-MEs	0.54 ± 0.06**	0.63	0.82 ± 0.05**	0.87
SYL3C/EP-MEs + SYL3C	0.61 ± 0.04**	0.71	0.88 ± 0.07**	0.93

**p < .01 *vs.* β-elemene. The IC_50_ of various formulations were calculated by the concentration of PTX, expect β-elemene group.

### Anticancer efficacy *in vivo*

**Figure 6.**
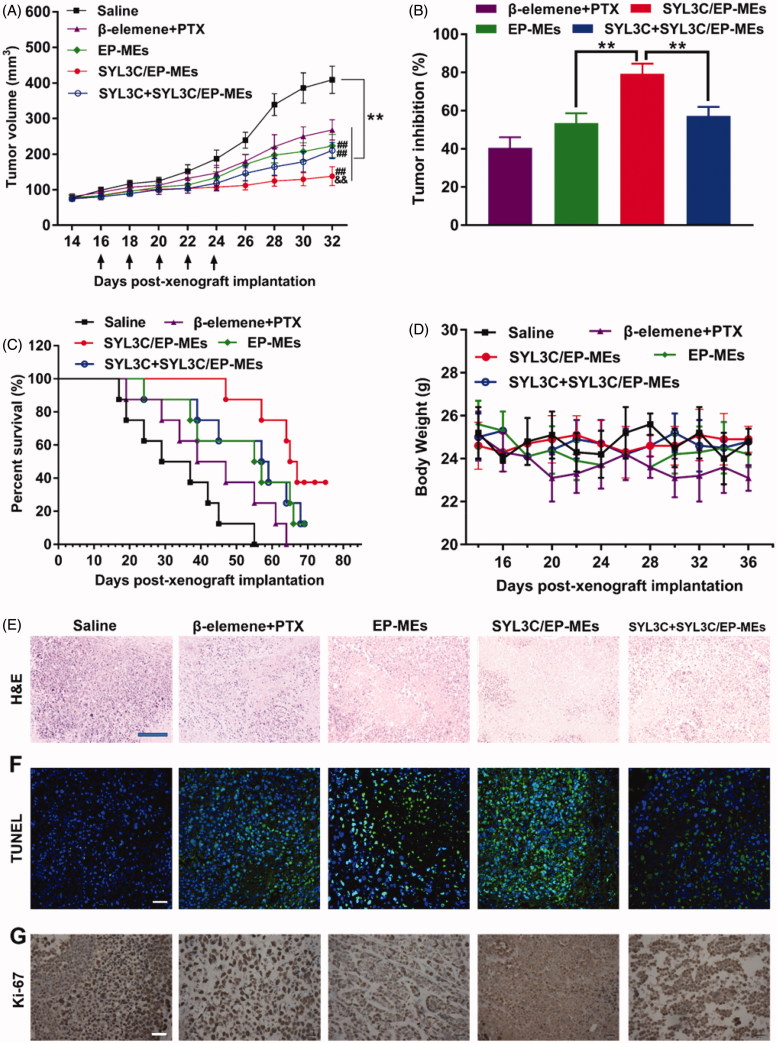
Antitumor efficacy in vivo. (A) Changes in tumor volume of mice treated with different formulations within 32 days post-xenograft implantation. *n* = 12, **p < .01 vs. saline; ^##^*p* < .01 vs. β-elemene + PTX; ^&&^*p* < .01 vs. EP-MEs and SYL3C + SYL3C/EP-MEs. (B) Inhibition of tumor growth of mice treated with different formulations. *n* = 12, **p < .01. (C) Survival period of mice treated with different formulations during 76 days of observation. *n* = 8. (D) Changes in body weight of mice treated with different formulations from day 14 to day 36 post-xenograft implantation. *n* = 12. (E) H&E staining, (F) TUNEL immunostaining and (G) immunohistochemical images of tumor slides of mice after different treatments. The bar is 100 μm.

Here, we investigated the tumor growth curve, tumor inhibition rate, survival rate, and pathological sections, during and after various treatments. As shown in Figure 6(A), tumor growth in the formulation-treated mice was significantly inhibited compared with that in the saline-treated group. On day 32, the tumor volume of SYL3C/EP-MEs-treated mice was significantly lower than that of mice in the EP-MEs and SYL3C + SYL3C/EP-MEs groups, verifying the necessity of the use of modified SYL3C aptamers for tumor-targeting anticancer treatment. At the end of the observation period, the tumor volume of mice in the EP-MEs group was significantly lower than that of mice in the β-elemene + PTX group, suggesting codelivery is critical to realize the synergistic effect. As expected, the tumor inhibition rate of mice in the SYL3C/EP-MEs group was 79.3 ± 5.2%, notably higher than that of animal models in the EP-MEs and SYL3C + SYL3C/EP-MEs groups (Figure 6(B)). SYL3C/EP-MEs treatment gained a high survival rate up to 37.5% at 76 days post treatment, whereas, only 12.5% of mice survived in EP-MEs and SYL3C + SYL3C/EP-MEs group. The maximum survival time of β-elemene + PTX-treated mice was 64 days (Figure 6(C)), suggesting that the modified tumor-targeting aptamer and intratumoral codelivery were necessary for prolonging the survival time of HT-29 tumor-bearing nude mice. Additionally, as exhibited in Figure 6(D), the body weight of mice decreased to some extent after β-elemene + PTX treatment, which may be related to strong PTX-induced immunosuppression, and liver, kidney, and gastrointestinal toxicity. However, there was no obvious weight loss after treatment with the other treatments. H&E staining revealed obvious tumor cell necrosis in the tumor sections of all treated mice (Figure 6(E)), with that of EP-MEs-treated mice being significantly larger than that of β-elemene + PTX-treated mice. The H&E stained sections of SYL3C/EP-MEs-treated mice showed the largest necrosis area compared with all other treatment groups, which could be attenuated by SYL3C pretreatment for 6 h. Considering the fact that free SYL3C aptamer at determined dosage barely had anticancer effect (Song et al., 2013), the treatment efficacy of SYL3C/EP-MEs was improved by SYL3C-led tumor targeting. The results of TUNEL immunofluorescence staining are displayed in Figure 6(F). Apoptotic cells labeled as the bright green fluorescence were significantly higher in the EP-MEs and SYL3C/EP-MEs groups, than in the β-elemene + PTX group, suggesting the successful induction of cell apoptosis by the 2 therapeutic drugs coloaded in the microemulsion system. The difference of fluorescence between the SYL3C/EP-MEs and SYL3C + SYL3C/EP-MEs group further validated the importance of the SYL3C aptamer as a tumor-targeted ligand. Further, Ki-67 is widely considered as a cell proliferation biomarker (Qu, 2018). We therefore investigated the presence of Ki-67 positive cells in various sections using the immunohistochemical staining method. As shown in Figure 6(G), the least brown areas were observed in the tumor sections of SYL3C/EP-MEs-treated mice, indicating that the tumor-targeted codelivery system significantly inhibited intratumoral cell proliferation, consistent with the antitumor efficacy results. Furthermore, the quantified results for TUNEL and Ki-67 sections were displayed in Figure S1, which was consistent with the corresponding qualified images.

### Safety evaluation* in vivo*

As previously reported, PTX causes immunosuppression, liver damage, and kidney toxicity, which is likely to lead to early chemotherapy termination (Campos et al., 2014). Here, we determined the safety of the microemulsion treatments by evaluating liver and kidney function, and hepatosplenic index. No significant changes were observed in the serum ALT, AST, BUN, and CREA levels of mice in all treatment groups at 12 h post treatment, compared with those of mice in the normal and saline groups, suggesting that liver and kidney function side effects of the administered treatments were limited by the administration doses and interval (Figure S2(A–D)). Figure S2(E,F) show that the liver and kidney indexes of the mice in each treatment group did not significantly fluctuate compared with those of the mice in the normal and saline groups.

### Mechanism of enhancement of anticancer efficacy

**Figure 7.**
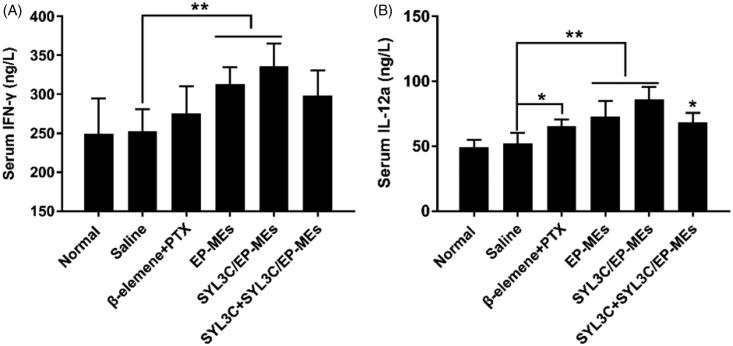
Serum level of (A) IFN-γ and (B) IL-12a of mice after 24 h of the last treatments. *n* = 4, *p < .05, **p < .01.

As previously reported, β-elemene could reduce the expression of mutant p53 and bcl-2 proteins, as well as increase macrophage clearance (Li et al., 2009). However, to date, the mechanism of the synergistic effect of β-elemene and PTX in a single delivery system remains unknown. M1 phenotype tumor-associated macrophages (TAMs) are well known to have strong tumor cell clearing abilities (Madeddu et al., 2018). To evaluate the influence of β-elemene on macrophage polarization, we quantified the serum levels of the two critical M1 specific phenotypic cytokines IFN-γ and IL-12a (Movahedi et al., 2010; Madeddu et al., 2018). As shown in Figure 7(A), the serum IFN-γ levels of EP-MEs- and SYL3C/EP-MEs-treated mice were significantly higher than those of saline-treated mice (**p < .05), but such phenomenon was not found in β-elemene + PTX-treated group. Likewise, the serum IL-12a levels of microemulsion-treated mice was significantly higher than those mice in the negative control group (**p < .05), suggesting that effective tumor-targeted delivery of β-elemene was favorable for the induction of M1 macrophage polarization (Figure 7(B)).

**Figure 8.**
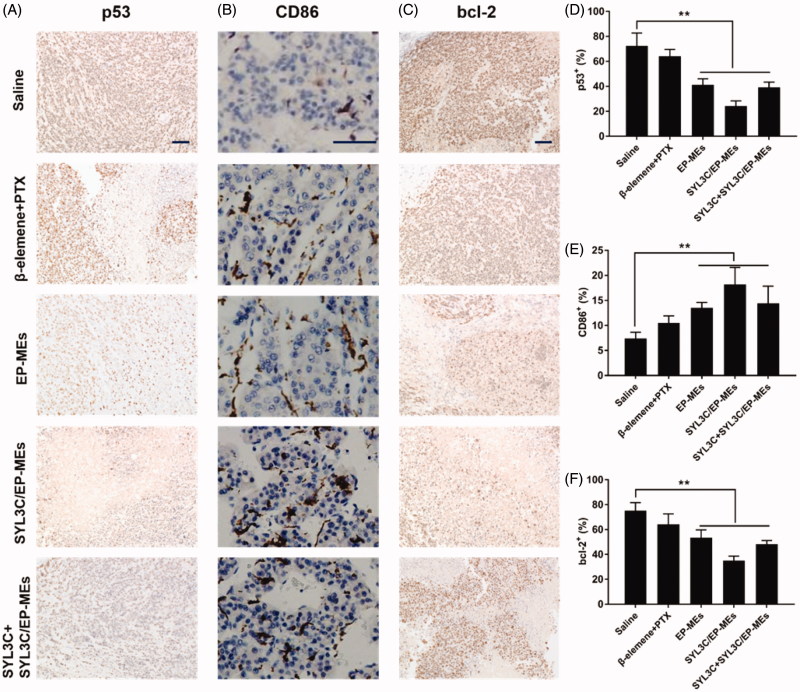
Immunohistochemical studies. Qualification of expression of (A) p53, (B) CD86 and (C) bcl-2 within the tumor tissues of mice after different treatments. Quantification of expression of (D) p53, (E) CD86 and (F) bcl-2 within the tumor tissues of mice after different treatments. *n* = 4, **p < .01. The bar is 100 μm.

Mutant p53 protein, a well-known tumor promoting factor, can eliminate the function of the wild-type p53 gene (Bodner et al., 1992). As shown in Figure 8(A,D), immunohistochemistry tumor sections of SYL3C/EP-MEs-treated mice showed the least amount of mutant p53 positive cells, probably due to sufficient β-elemene accumulation in the tumor sites. To add, CD86 is a widely used M1 TAMs characteristic marker (Dong et al., 2016). As shown in Figure 8(B,E), the brown areas in the immunohistochemical images of tumor sections of the SYL3C/EP-MEs-treated mice were significantly larger than those of the saline-treated mice. However, CD86 expression was not notably higher in the β-elemene + PTX-treated mice, further proving the importance of precise β-elemene delivery to the tumor sites. Further, bcl-2 protein can inhibit the induction of apoptosis and promote cell survival (Strasser et al., 1995; Li et al., 2009). Figure 8(C,F) show that the amount of bcl-2 positive cells in the tumor sections of microemulsion-treated mice were significantly lower than those in the tumor sections of the saline-treated mice. However, bcl-2 expression was not decreased in the β-elemene + PTX group, further validating our hypothesis that codelivery of both two drugs and precise tumoral accumulation synergistically contributed to the enhanced anticancer effect.

## Conclusion

In this work, we have prepared a β-elemene and PTX-coloaded microemulsion anchored with SYL3C aptamer, which is capable of targeting the EpCAM over-expressed colorectal cancer cells and enhancing the anti-colorectal treatment. SYL3C/EP-MEs have high encapsulation efficiency for both contents and are able to release in a manner of pH-sensitivity, which is helpful to elevate apoptosis rate and cytotoxicity against HT-29 cells. In the HT-29 tumor xenograft-bearing nude mice model studies, SYL3C/EP-MEs inhibit the tumor growth, prolong the survival period of mice, and promote the cell apoptosis within the tumor tissues. The underlying mechanisms of enhanced anticancer efficacy in vivo are associated with the induction of M1 macrophage polarization, the downregulation of mutant p53 protein and the reduction of bcl-2 protein expression. In summary, this paper provides a promising approach to the combinational colorectal-targeted therapy.

## Supplementary Material

Supplemental Material
