# Supplementary material for: SYL3C aptamer-anchored microemulsion co-loading β-elemene and PTX enhances the treatment of colorectal cancer
Source: Drug Deliv. 2019 Sep 14;26(1):886–97. doi: 10.1080/10717544.2019.1660733 (PMC6758640; doi:10.1080/10717544.2019.1660733)
Supplement: Supplemental Material [file IDRD_A_1660733_SM8634.docx]

**Supplemental information**

**SYL3C aptamer-anchored microemulsion co-loading β-elemene and PTX enhances the treatment of colorectal cancer**

Xiaorong Zhou^1,#^, Chuanpei Cao^2,#^, Nan Li^1^, Shaofei Yuan^1,3*^

^1^ Department of Medicine, Jiangsu Cancer Hospital, Nanjing 210009, People’s Republic of China

^2^ Gastrointestinal Surgery, Affiliated Hospital of Jiujiang University, Jiujiang 332000, People’s Republic of China

^3^ Department of Medical Oncology, The Third Affiliated Hospital of Wenzhou Medical University, Wenzhou 325000, People’s Republic of China

^*^ Correspondence to: Shaofei Yuan, Tel: +86 577 65866342; Fax: 86 577 65866342; E-mail: ysf1004@163.com; Address: Wansong Road 108^#^, Ruian City, Zhejiang province, People’s Republic of China

**^#^** These authors contributed equally to this work

**
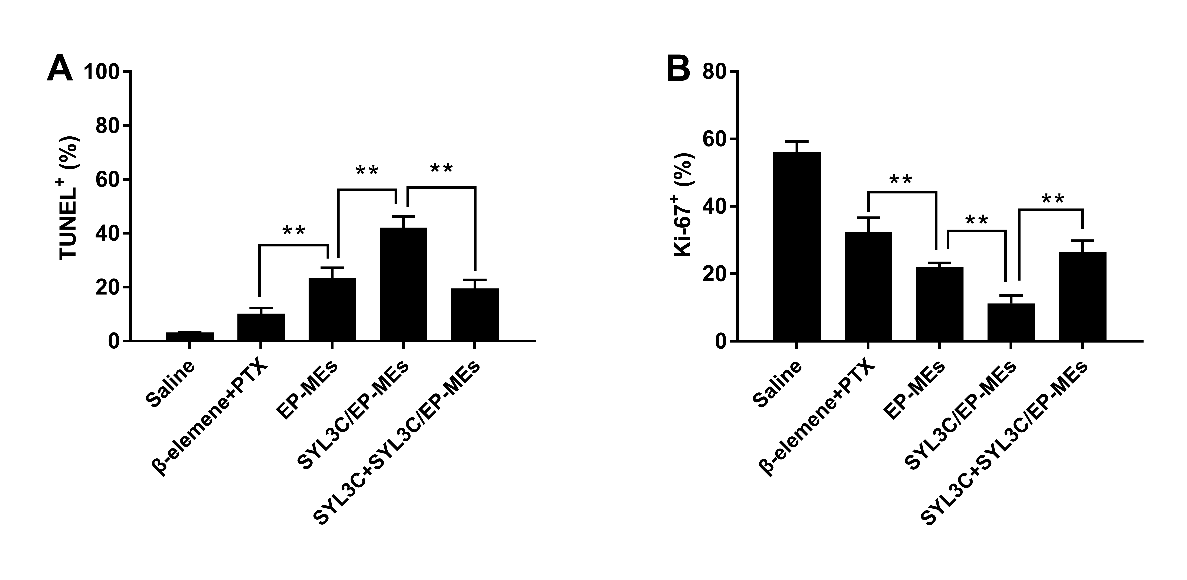
**

Figure S1. Quantified results for (A) TUNEL (immunofluorescence) and (B) Ki-67 (immunohistochemical) sections after different treatments. n = 4.


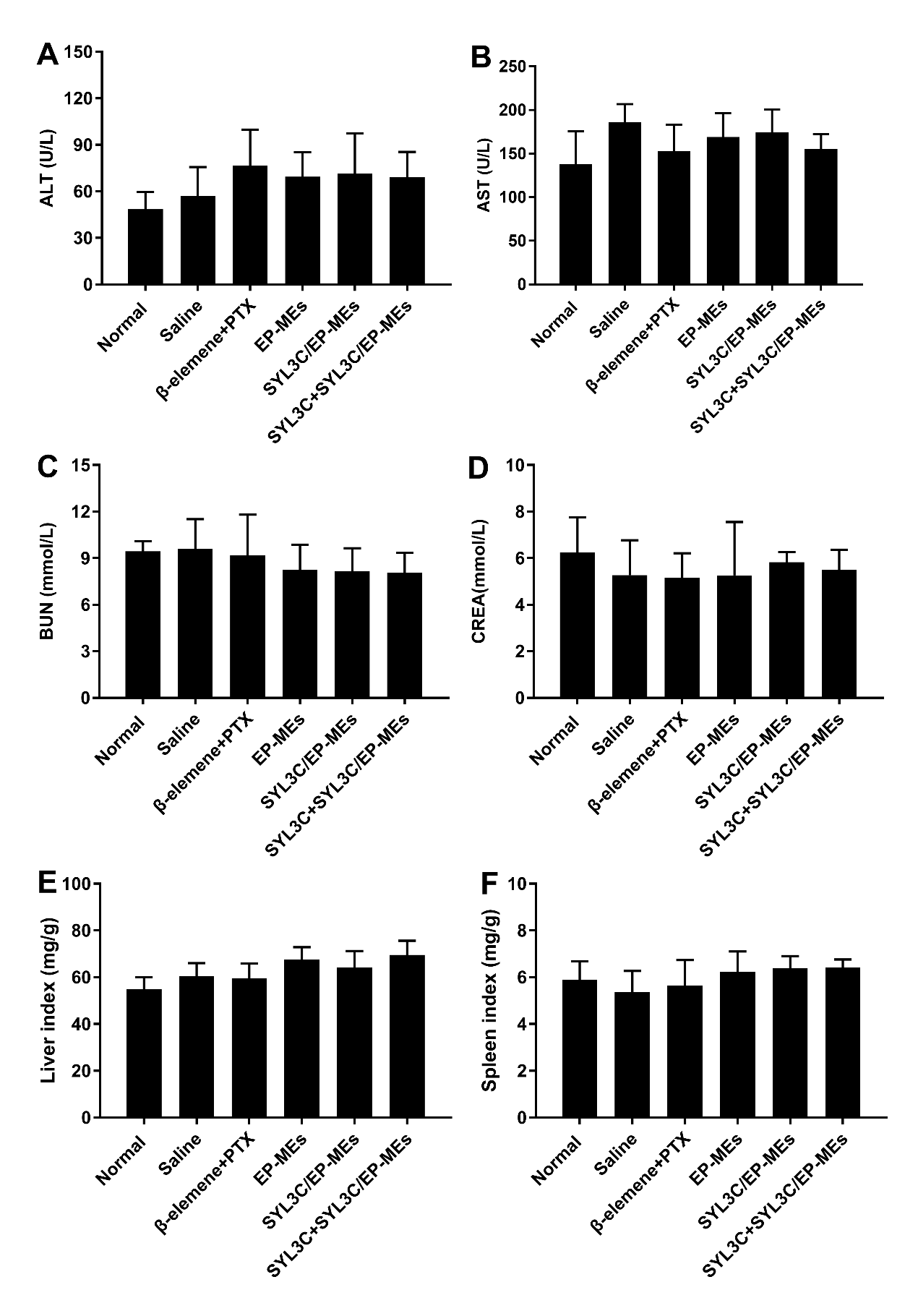


Figure S2. Safety evaluation on treatments. Serum level of (A) ALT, (B) AST, (C) BUN and (D) CREA of mice after 12 h of the last administration. (E) Liver and (F) spleen index of mice at the end of the observation. n = 4.
